# Convergence of endobariatrics and endohepatology for evaluation and treatment of obesity and nonalcoholic fatty liver disease

**DOI:** 10.1055/a-2094-9794

**Published:** 2023-06-27

**Authors:** Arunkumar Krishnan, Sardar Momin Shah-Khan, Yousaf Hadi, Shyam Thakkar, Shailendra Singh

**Affiliations:** Section of Gastroenterology and Hepatology, West Virginia University School of Medicine, Morgantown, West Virginia, United States

Endohepatology is an emerging field that utilizes diagnostic and therapeutic endoscopic ultrasound (EUS) to manage liver disease. In recent years, endoscopic sleeve gastroplasty (ESG) has emerged as a safe and effective approach to treating obesity and nonalcoholic fatty liver disease (NAFLD) 1 . We present a combined approach of endobariatrics and endohepatology with the concept of a “one-stop shop” to evaluate portal hypertension, obesity, and associated NAFLD treatment.

A 53-year-old woman with a body mass index of 47.4 kg/m ^2^ and NAFLD was referred for evaluation for ESG. The patient was previously denied bariatric surgery after screening endoscopy revealed features of esophageal varicosities ( Fig. 1 ). A decision was made to perform an assessment of portal hypertension and obtain a liver biopsy prior to ESG.

**Fig. 1:**
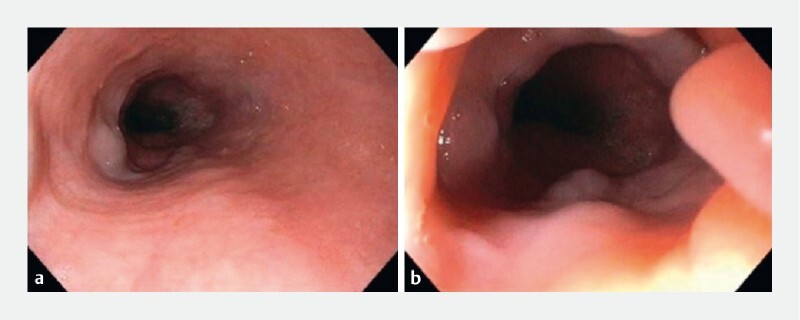
Upper endoscopy. **a, b** Lesions resembling several small-sized varices were seen in the distal esophagus.

EUS-guided portal pressure measurement directly measures the portal pressure gradient ( Fig. 2 , Fig. 3 , Video 1 ) 2 . A transgastric, transhepatic puncture with a 25-gauge fine-needle aspiration needle equipped with a compound manometer (Cook Medical, Bloomington, Indiana, USA) was performed into the middle hepatic vein, and a portal vein was identified in order to measure the portal vein pressure. Table 1 presents the portal pressure measurements. A liver sample from the left lobe was obtained via a transgastric puncture using a 19-gauge fine-needle biopsy needle, and pathology demonstrated moderate macro- and microsteatosis without significant fibrosis ( Video 1 ).

**Fig. 2:**
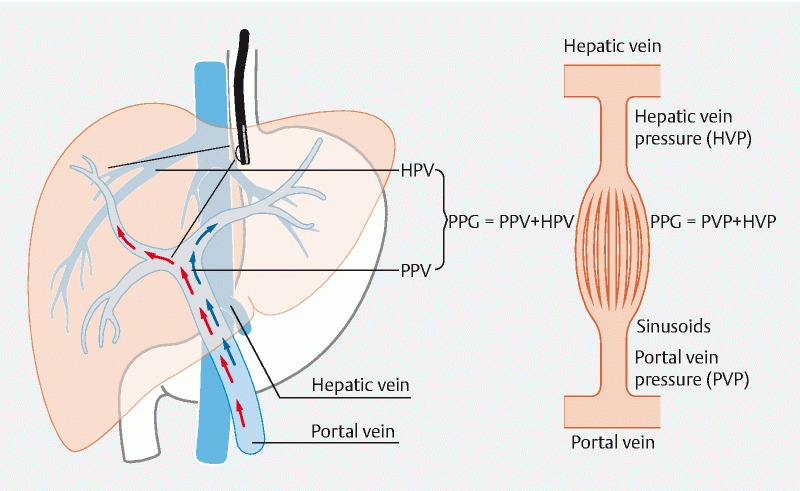
Endoscopic ultrasound-guided portal pressure gradient (PPG) measurement. The PPG represents the difference between direct portal vein pressure and hepatic vein pressure. HVP, hepatic vein pressure; PVP, portal vein pressure.

**Fig. 3:**
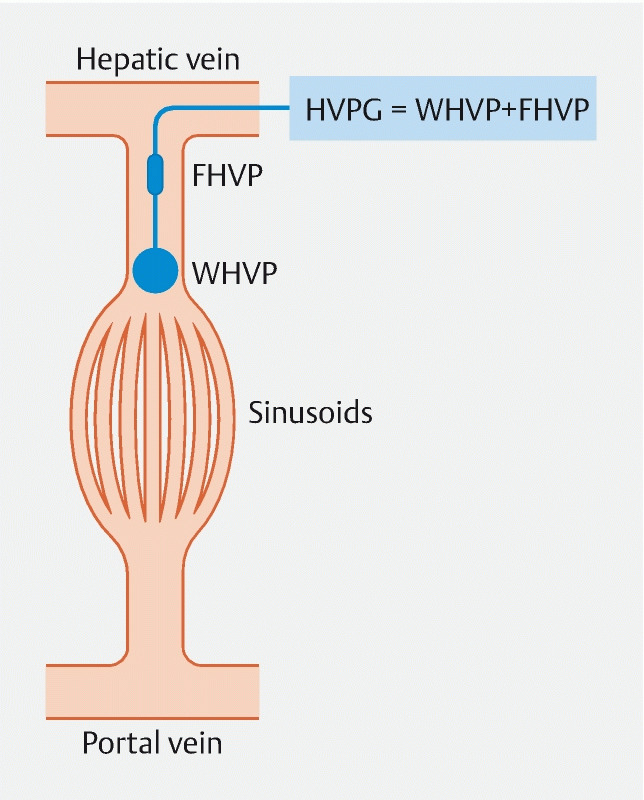
Transjugular hepatic venous pressure gradient (HVPG) measurement. The HVPG is the difference between the wedged and the free hepatic venous pressures. HVPG represents the gradient between pressures in the portal vein and the intra-abdominal portion of the inferior vena cava. FHVP, free hepatic venous pressure; WHVP, wedged hepatic venous pressure.

**Video 1** Endoscopic ultrasound-guided portal pressure measurement and liver biopsy to evaluate nonalcoholic fatty liver disease, and endoscopic sleeve gastroplasty to treat class III obesity.


**Table 1:** Endoscopic ultrasound-guided portal pressure gradient measurements.

	Venous pressure measurements, mmHg			
	1	2	3	Mean
Hepatic vein	10	12	10	10.6
Portal vein	14	14	14	14
Portal pressure gradient				3.4

Argon plasma coagulation was used to mark and induce de-epithelialization to promote tissue apposition 3 . A ‘U’ pattern was adopted using an endoscopic suturing device (OverStitch; Apollo Endosurgery, Inc., Austin, Texas, USA), starting at the incisura-anterior surface, followed by the greater curvature and then the posterior wall ( Fig. 4 , Video 1 ).

**Fig. 4:**
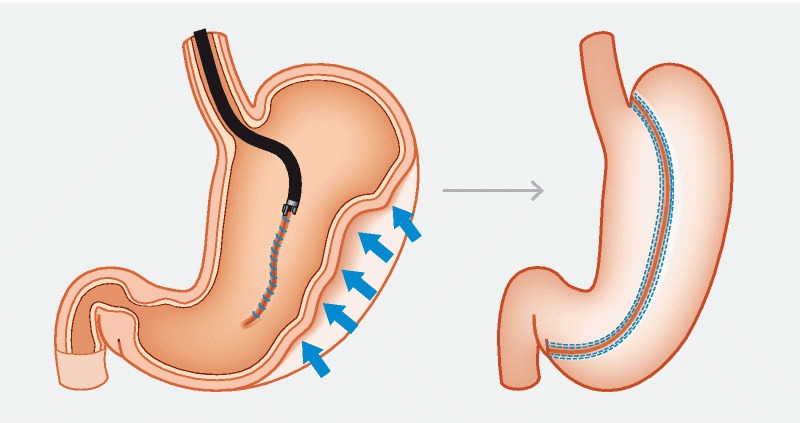
An illustration of the creation of the endoscopic sleeve gastroplasty.

At 1-year follow-up, the total weight loss percentage was 27 %, and FibroScan (Echosens, Paris, France) showed no steatosis or fibrosis.

Our case demonstrates the successful convergence of endohepatology and endobariatric techniques for evaluating portal hypertension with the measurement of portal pressure, diagnosis of NAFLD with EUS-guided liver biopsy, and treatment of obesity with ESG. These procedures work in parallel, allowing for a comprehensive, one-stop approach to diagnosing and managing NAFLD and obesity.

Endoscopy_UCTN_Code_TTT_1AU_2AF

## Notes

Endoscopy E-Videos

https://eref.thieme.de/e-videos
*E-Videos* is an open access online section of the journal *Endoscopy* , reporting on interesting cases and new techniques in gastroenterological endoscopy. All papers include a high-quality video and are published with a Creative Commons CC-BY license. Endoscopy E-Videos qualify for HINARI discounts and waivers and eligibility is automatically checked during the submission process. We grant 100% waivers to articles whose corresponding authors are based in Group A countries and 50% waivers to those who are based in Group B countries as classified by Research4Life (see: https://www.research4life.org/access/eligibility/ ). This section has its own submission website at https://mc.manuscriptcentral.com/e-videos
